# Where There Is Asbestos, There Is Mesothelioma: Filling in the Data Blanks

**DOI:** 10.1289/ehp.119-a177b

**Published:** 2011-04

**Authors:** Rebecca Clay Haynes

**Affiliations:** **Rebecca Clay Haynes** has written for *EHP* since 1993. Her work has also appeared on National Public Radio and in the *Christian Science Monitor* and *The Environmental Forum*. In addition, she is the author of two children’s science books related to astronomy and space exploration

Malignant mesothelioma is caused almost exclusively by exposure to asbestos, and countries that have used asbestos nearly always have cases of mesothelioma. Tracking the disease has proved difficult, however, because not all developing countries that use asbestos collect mesothelioma incidence data. In a new global estimate of unreported mesothelioma, researchers predict that at least one case of disease goes unreported for every four to five known cases worldwide **[*EHP*** **119(4):514–518; Park et al.]**.

The authors compared cumulative asbestos use from the U.S. Geological Study with disease cases reported to the World Health Organization. Because symptoms of mesothelioma often appear decades after exposure, the authors examined the relationship between the 15-year cumulative number of reported mesothelioma cases during 1994–2008 and cumulative asbestos use during 1920–1970 among countries with data on both mesothelioma and asbestos use. The resulting relationship helped them predict the number of unreported mesothelioma cases in countries providing information on asbestos use but not on mesothelioma.

The authors found that cumulative asbestos use in 89 countries totaled more than 65 million metric tons during 1920–1970. Of the 56 countries also reporting mesothelioma data, there were more than 174,000 estimated cases and 92,000 reported deaths during 1994–2008 (most mesothelioma patients succumb to the disease shortly after diagnosis, so numbers of new cases are very similar to numbers of deaths from the disease). When extrapolating these data to the 33 countries not reporting mesothelioma, the authors estimated an additional 39,000 cases would have occurred during that same 15-year period.

This estimate is conservative, say the authors, and they warn that because asbestos has a long industrial life span, and its use has quintupled since 1970, many countries should anticipate a higher disease burden in the years to come. The new study does not account for this 40-year increase.

The authors propose that developed countries share their experience and technology to help developing countries better diagnose, report, and manage cases of mesothelioma. They also argue that all countries should move toward a complete ban on asbestos—although the long latency period means mesothelioma deaths would continue for decades, the disease would eventually disappear as asbestos use is phased out and exposure is eventually eliminated.

## Figures and Tables

**Figure:**
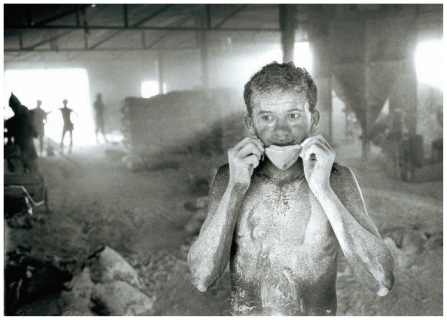
An asbestos factory in Ipubi, Pernambuco, Brazil, date unknown. Brazil has now banned asbestos production in this state, but mesothelioma’s long latency period means cases will continue to emerge for decades.

